# Dr. Henry Fraser Campbell

**Published:** 1892-01

**Authors:** 


Dr. Henry Fraser Campbell, of Augusta, Ga., died at his home on Tuesday, December 15, 1891, in the sixty-eighth year of his age. He graduated in medicine in 1842, and settled in Augusta the same year, where, except during the period of the war and two Winters spent in New Orleans, he has remained and practised his profession.

His earlier achievements were made as a general surgeon, but for many years he has been distinctly known as a gynecologist. Dr. Campbell has been an extensive and voluminous writer, and has contributed some of the most valuable papers to the literature of surgery and gynecology of any man in modern times. His fame as a teacher and writer extended beyond his own country, and in Europe his name was almost as well known as among his colleagues at home. He was a member of all the prominent societies in his State. He was president of the American Medical Association in 1885, and has been for some years an active member of the American Gynecological Association, and still later of the Southern Surgical and Gynecological Association.

He was a man of great amiability of character, a genial companion, whose face was always a gleam of sunshine, and who lent himself heartily to the promotion of health, happiness and longevity wherever his ministrations were sought or rendered. He leaves a family who were devoted to him both as husband and father, and for whom we express our tenderest sympathy.

